# Assessment of radiographic and periodontal status among obese patients with and without type-2 diabetes and systemically healthy subjects

**DOI:** 10.2340/aos.v84.44957

**Published:** 2025-12-15

**Authors:** Marwa Y. Shaheen, Amani M. Basudan, Abeer S. Alzawawi, Fatemah M. AlAhmari, Nouf Alshibani, Reem Al-kattan, Lamees Alssum, Fatima Alzahraa Yassin Shaheen, Arwa Ameen Talakey

**Affiliations:** a Department of Periodontics and Community Dentistry, College of Dentistry, King Saud University, Riyadh, P.O. Box.2455, Saudi Arabia; b Consultant, Emergency Department, SFHPR security force hospital program, Riyadh, Saudi Arabia

**Keywords:** Alveolar bone loss, body mass index, obesity, type-2 diabetes mellitus, probing depth

## Abstract

**Objective:**

The objective was to assess the radiographic and periodontal status among obese patients with and without type-2 diabetes and systemically-healthy subjects.

**Methods:**

Participants were divided into three groups: (a) Non-diabetic obese patients; (b) Type-2 diabetic obese patients; and (c) self-reported systemically healthy controls. Demographic data, daily oral hygiene maintenance protocols and education status (ES) were collected using a questionnaire. Information regarding family history of diabetes mellitus and obesity was recorded. Hemoglobin A1c (HbA1c) levels and body mass index (BMI) were recorded. Plaque index (PI), bleeding-on-probing (BoP), probing depth (PD), clinical attachment loss (CAL), marginal bone loss (MBL) and number of missing teeth (MT) were recorded. Group comparisons were done, and the correlation between BMI, HbA1c and periodontal parameters was assessed using linear regression models. Level of significance was set at *P* < 0.05.

**Results:**

Sixty-three individuals (21 non-diabetic obese, 22 type-2 diabetic obese and 20 systemically healthy controls) were included. Percentages of sites that demonstrated plaque (*P* < 0.05) and BoP (*P* < 0.05), and scores of CAL (*P* < 0.05), PD (*P* < 0.05), MBL (*P* < 0.05) and MT (*P* < 0.05) were higher among non-diabetic obese and type-2 diabetic obese patients than controls. There was no difference in these parameters among type-2 diabetic and non-diabetic obese patients. There was a significant correlation between HbA1c (*P* < 0.05) and BMI (*P* < 0.05) and PD in type-2 diabetic and non-diabetic obese patients. There was no correlation between HbA1c and BMI in all groups.

**Conclusions:**

Periodontal tissue destruction is more pronounced in obese individuals, irrespective of diabetic status. This suggests that excess adiposity plays a central role in periodontal breakdown, regardless of diabetic status.

## Introduction

The etiopathogenesis of periodontal diseases, including periodontitis, is multifactorial. Inadequate adherence to routine oral hygiene maintenance (OHM) protocols, such as toothbrushing and flossing is the most common risk factor of periodontal inflammation [1, 2]; however, evidence from scientific literature has shown that a compromised systemic health status (immunosuppression) also contributes to the etiology and progression of periodontal diseases [3–5]. Studies [6–8] have shown that scores of plaque index (PI), bleeding on probing (BoP), probing depth (PD), clinical attachment loss (CAL) and marginal bone loss (MBL) are worse among patients with poorly-controlled type-2 diabetes mellitus (DM) than non-diabetic individuals and type-2 diabetic patients with well-controlled glycemic levels. Persistent hyperglycemia is a hallmark of poorly-controlled DM [6]; and significantly impacts periodontal health by exacerbating tissue destruction and inflammation. Elevated blood glucose levels lead to increased production of reactive oxygen species (ROS), resulting in oxidative stress within periodontal tissues [9]. This oxidative stress damages cellular components, including lipids, proteins, and DNA, thereby impairing the function and integrity of periodontal structures [9]. Moreover, hyperglycemia promotes the formation of advanced glycation end-products (AGEs) through non-enzymatic reactions between excess sugars and amino groups in proteins and nucleic acids. Advanced glycation end-products accumulate in periodontal tissues, altering cellular functions and triggering inflammatory responses [10, 11]. The interaction between AGEs and their receptors (RAGE) on cell surfaces further amplifies oxidative stress and inflammation, creating a detrimental cycle that accelerates periodontal tissue breakdown [12].

Bidirectional relation between type-2 DM and obesity (individuals with a body mass index [BMI] of at least 30 Kg/m^2^) is well-established, with each condition exacerbating the other through complex metabolic, inflammatory, and hormonal mechanisms [13–15]. Excess adipose tissue, particularly visceral fat, induces insulin resistance through the release of pro-inflammatory cytokines such as tumor necrosis factor-alpha (TNF-α) and interleukin-6 (IL-6) [16, 17]. Such proinflammatory cytokines impair insulin signaling and contribute to chronic low-grade inflammation, a key feature in the pathogenesis of type-2 DM [18]. Results from a recent study based on data from the National Health and Nutrition Examination Survey showed that an increased BMI is a risk factor of periodontitis [19]. It has also been reported that outcomes of non-surgical periodontal interventions (such as scaling and root planing [SRP]) are compromised among obese than normal weight subjects with periodontitis [20]. A critical review of indexed databases showed that there are no studies that have compared the clinical and radiographic periodontal status among obese patients with and without type-2 DM.

The objective of the present observational study was to compare the clinical and radiographic periodontal status among obese patients with and without type-2 DM and controls (systemically healthy controls). The objective was to assess the radiographic and periodontal status among obese patients with and without type-2 diabetes and systemically healthy subjects.

## Materials and methods

### Study design and guidelines

The present observational study was performed in accordance with the Strengthening the Reporting of Observational Studies in Epidemiology (STROBE) guidelines to ensure methodological transparency and comprehensive reporting of study design, data collection, analysis, and interpretation [21]. Through a convenience sampling (CS) approach, participants presenting to the dental clinics of the College of Dentistry, King Saud University, Riyadh, Saudi Arabia, were included.

### Ethical approval

The study protocol received ethical approval from the Research Ethics Review Committee of the College of Dentistry, King Saud University, Riyadh, Saudi Arabia (Approval No. E-25-9746). Participation was entirely voluntary, and all individuals who chose to participate were provided with an information sheet in both English and Arabic, outlining the study’s purpose in clear and accessible language. The information sheet explicitly stated that participation was voluntary and that non-participation or withdrawal would not result in any penalties. Participants were encouraged to ask questions, and those who consented to participate in the study were required to review and sign a written informed consent form.

### Inclusion and exclusion criteria

The inclusion criteria were as follows: (a) individuals aged at least 18 years old; (b) Patients with medically diagnosed type-2 DM (hemoglobin A1c [HbA1c] levels ≥ 6.5%) [22]; (c) patients with medically diagnosed obesity (BMI ≥ 30 kilograms per square meters [Kg/m^2^]) [23] (d) self-reported systemically healthy individuals (controls) [6]. The exclusion criteria were as follows: (a) patients with self-reported systemic diseases other than type-2 DM and obesity, such as cardiovascular and renal disorders, hepatic disorders, psychiatric diseases and viral infections including HIV/AIDS; (b) self-reported combustible and non-combustible nicotinic product users and alcohol users; (c) lactating and/or pregnant females; (d) grossly carious teeth and third molars; (e) completely edentulous individuals; (f) patients with a history of bariatric surgery; (g) individuals that reported to have used antibiotics, steroids, non-steroidal anti- inflammatory drugs and/or surgical/non-surgical periodontal interventions within the past 90 days.

### Study groups and duration

Participants were divided into the following groups: (a) non- diabetic obese patients; (b) type-2 diabetic obese patients; and (c) self-reported systemically healthy individuals. The study was performed between May 2024 and February 2025.

### Questionnaire

A structured questionnaire was administered to all participants to collect demographic, medical, and oral hygiene-related information. Participants provided details regarding their age, sex, duration of obesity and/or type-2 DM, and family history of obesity and diabetes. The questionnaire gathered information about educational status was also collected. Individuals that reported to have attained education up to the 10^th^ and 12^th^ grades were classified as having achieved school- and college-level education, respectively [24]. Individuals who reported to have graduated from a University were categorized as having university-level education [24]. Information on toothbrushing (once or twice daily, sometimes or never) and flossing (once or twice daily, sometimes or never) was collected. Participants were also asked whether they visited a dentist or dental hygienist biannually, annually, only in cases of pain or discomfort, or not at all.

### Assessment of body mass index

In the present observational study, the BMI was measured as described elsewhere [25]. In summary, the BMI was measured by recording each participant’s weight and height using a calibrated digital scale (Jadssox Lcxliga Height And Weight Scale, Beijing, China) and a stadiometer (Jadssox Lcxliga Height And Weight Scale, Beijing, China), respectively. Weight was measured in Kg with participants wearing light clothing and no shoes, and height was recorded in meters with the participant standing upright against a vertical surface. Body mass index was then calculated using the standard formula: BMI = weight (Kg) ÷ height (m^2^). The BMI was measured and recorded by a trained and calibrated investigator (*Kappa* score 0.88) who was blinded to the glycemic status of the participants.

### Assessment of hemoglobin A1c

In all patients, the hemoglobin A1c (HbA1c) levels were measured by a trained and calibrated investigator (*Kappa* score 0.9) using a commercially available digital kit (PTS Diagnostics, A1CNow^®^ + system, Whitestown, IN, USA). The commercial kit was used as described in previous investigations [26, 27].

### Clinical periodontal examination

In all patients, full mouth PI, BoP, PD, CAL and number of missing teeth (MT) were recorded. The PI [28] and BoP [29] were recorded on four surfaces (mesial, distal, buccal/facial and palatal/lingual) per tooth and presented as percentages of sites that demonstrated these parameters. Sites that exhibited the presence of dental plaque and/or BoP were assigned a score of ‘1’, whereas sites that did not demonstrate these parameters were assigned a score of ‘0’. The PD [30, 31] and CAL were recorded to the nearest millimeter using a graded probe (Hu-Friedy, Chicago, IL, USA) on six surfaces per tooth (mesiobuccal/facial, midbuccal/facial, distobuccal/facial, mesiolingual/palatal, midlingual/palatal and distolingual/palatal). All clinical measurements were recorded to the nearest millimeter. All clinical and radiographic assessments were performed by a trained and calibrated examiner (MYS; *Kappa* score 0.86) who was blinded to the glycemic status of the participants.

### Assessment of marginal bone loss

The MBL was assessed on the mesial and distal surfaces of all teeth by a trained and calibrated investigator (Kappa score 0.86). The MBL was measured in millimeters on digital intra-oral radiographs taken using the long-cone paralleling technique. The MBL was defined as the vertical distance from two-millimeters below the cementoenamel junction to the alveolar crest [6].

### Classification of periodontitis

In all participants, periodontitis was classified using the criteria described in the ‘Consensus report of workgroup 2 of the 2017 World Workshop on the Classification of Periodontal and Peri-Implant Diseases and Conditions’ [32].

### Sample-size estimation

Sample size estimation was performed using the G*Power 3.1 software (University of Düsseldorf, Düsseldorf, Germany). The primary and secondary outcome variables were CAL and BoP, respectively; and the calculation was based on an analysis of variance (ANOVA) model assuming an alpha level of 5% and a power of 80%. It was estimated that a minimum of 18 individuals per group was determined to be necessary to achieve sufficient power for detecting statistically significant differences in CAL among the study groups.

### Statistical analysis

Statistical analysis was done using computer software (IBM, SPSS, Version 22, Armonk, NY., USA). The primary outcome variables were radiographic and clinical periodontal inflammatory parameters (PI, BoP, CAL, PD and MBL). Data were assessed for normality using the Shapiro-Wilk test. Demographic data collected through the questionnaire were summarized using mean ± standard deviation (SD) for continuous variables and frequencies (percentages) for categorical variables. Differences among the three groups (Type-2 diabetic obese, non-diabetic obese, and controls) were analyzed using the ANOVA or Kruskal-Wallis test, depending on data normality, for continuous variables. Correlation between the secondary outcome variables (HbA1c and BMI) and PD was assessed using Pearson’s correlation test. A *p*-value < 0.05 was considered statistically significant.

## Results

### General characteristics of the study cohort

**Figure 1:**
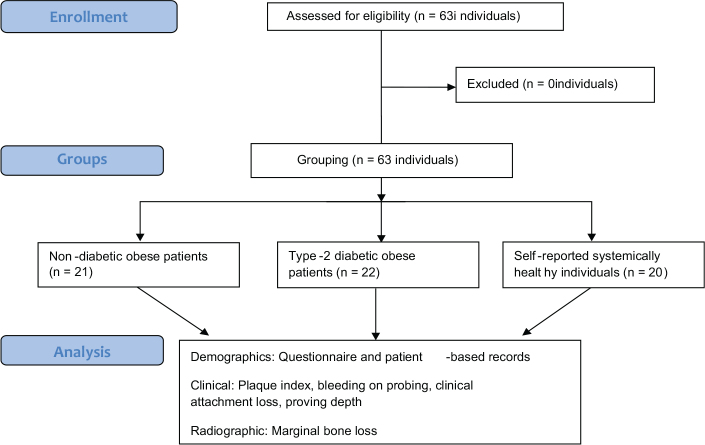
STROBE flow diagram.

In total, 63 individuals (21 non-diabetic obese, 22 type-2 diabetic obese and 20 systemically healthy controls) were included (Figure 1). The mean age was comparable across groups: 52.4 ± 7.2 years (non-diabetic obese), 50.5 ± 2.8 years (type-2 diabetic obese), and 53.1 ± 2.9 years (controls). In all groups, most of the participants were male. Non-diabetic obese patients had obesity for an average of 10.1 ± 0.8 years, while type-2 diabetic obese patients had it for 8.8 ± 0.4 years. The latter group also had DM for an average of 7.4 ± 1.5 years. A family history of obesity was reported by 33.3% and 40.9% of non-diabetic and type-2 diabetic obese patients, respectively. A family history of DM was reported by 47.6% of non-diabetic obese and 54.5% of the type-2 diabetic obese patients and none of the controls. The mean BMI was significantly higher among non-diabetic (*P* < 0.05) and type-2 diabetic obese patients (*P* < 0.05) than controls. The mean HbA1c levels were significantly higher among type-2 diabetic obese individuals (*P* < 0.05) than non-diabetic obese patients and controls. The majority of non-diabetic obese and diabetic obese patients brushed their teeth once daily, whereas 85% of systemically healthy controls brushed twice daily. Seventy-five percent of the controls reported to have attained university-level education compared to non-diabetic (14.3%) and type-2 diabetic obese (13.6%) patients. None of the obese patients (type-2 diabetic or non-diabetic) reported flossing, whereas 45% of the systemically healthy subjects flossed occasionally. Fifty percent of the systemically healthy controls reported having visited a dentist/dental hygienist within the past 12 months, and 78.9% and 61.1% of non-diabetic obese and type-2 diabetic obese patients, respectively, sought dental care when necessary. Among non-diabetic obese and type-2 diabetic obese individuals, 21.1% and 38.9% individuals, respectively reported having never visited a dentist/dental hygienist (Table 1). According to dental records, patients diagnosed with type-2 DM had been prescribed oral anti-hyperglycemic medications, provided with dietary counseling, and advised to engage in regular physical exercise for the management of their glycemic status.

**Table 1:** Characteristics of the study cohort.

Parameters	Non-diabetic obese patients	Type-2 diabetic obese patients	Systemically healthy individuals
Participants (*n*)	21	22	20
Mean age in years	52.4 ± 7.2 years	50.5 ± 2.8 years	53.1 ± 2.9 years
Sex (Male:Female)	14:5	14:4	12:8
Duration of obesity	10.1 ± 0.8 years	8.8 ± 0.4 years	NA
Duration of type-2 DM	NA	7.4 ± 1.5 years	NA
Family history of obesity	7 (33.3%)	9 (40.9%)	NA
Family history of DM	10 (47.6%)	12 (54.5%)	NA
Body mass index in Kg/m^2^	32.6 ± 1.2 Kg/m^2*^	32.8 ± 0.7 Kg/m^2*^	19.7 ± 1.2 Kg/m^2^
Hemoglobin A1c levels (%)	5.1 ± 0.06^†^	10.8 ± 0.2	5 ± 0.08^†^
Education status			
School-level	12 (57.1%)	15 (68.2%)	NA
College-level	6 (28.6%)	4 (18.2%)	5 (25%)
University-level	3 (14.3%)	3 (13.6%)	15 (75%)
Toothbrushing			
Once daily	16 (76.2%)	16 (72.7%)	3 (15%)
Twice daily	3 (14.3%)	6 (27.3%)	17 (85%)
Sometimes	2 (9.5%)	NA	NA
Never	NA	NA	NA
Flossing			
Once daily	NA	NA	NA
Twice daily	NA	NA	NA
Sometimes	NA	NA	9 (45%)
Never	19 (100%)	18 (100%)	11 (55%)
Most recent visit to a dentist			
Within 6 months	NA	NA	NA
Six to 12 months ago	NA	NA	NA
Over a year ago	NA	NA	10 (50%)
Only when necessary	17 (80.9%)	15 (68.2%)	10 (50%)
Never	4 (19.1%)	7 (31.8%)	NA

DM: Diabetes mellitus; Kg/m^2^: Kilograms per square meters; NA: Not applicable.

* Compared with systemically healthy controls (*P* < 0.05).

† Compared with type-2 diabetic obese patients (*P* < 0.05).

### Clinical and radiographic periodontal parameters

The percentages of sites that demonstrated plaque and BoP (*P* < 0.05) were significantly higher among non-diabetic and type-2 diabetic obese patients compared with systemically healthy controls. The scores of CAL (*P* < 0.05), PD (*P* < 0.05), mesial (*P* < 0.05) and distal (*P* < 0.05) MBL and numbers of MT (*P* < 0.05) were significantly higher among non-diabetic and type-2 diabetic obese patients compared with systemically healthy controls. There was no statistically significant difference in the scores of PI, BoP, PD, CAL, mesial and distal MBL and numbers of MT among type-2 diabetic and non-diabetic obese patients (Table 2). All patients had Stage-II Grade B periodontitis.

**Table 2:** Clinical and radiographic periodontal status of participants.

Parameters	Non-diabetic obese patients	Type-2 diabetic obese patients	Systemically healthy controls
Plaque index (% of sites)	82.3 ± 3.1%^*^	80.6 ± 1.4%^*^	20.4 ± 3.3%
Bleeding on probing (% of sites)	85.1 ± 2.7%^*^	83.2 ± 2.2%^*^	12.1 ± 1.8%
Probing depth (in mm)	5.5 ± 0.3 mm^*^	6.2 ± 0.07 mm^*^	2.3 ± 0.04 mm
Clinical attachment loss (in mm)	3.3 ± 0.1 mm^*^	3.2 ± 0.08 mm^*^	0.7 ± 0.04 mm
Marginal bone loss (mesial) in mm	4.8 ± 0.3 mm^*^	5.1 ± 0.08 mm^*^	1.1 ± 0.2 mm
Marginal bone loss (distal) in mm	5.05 ± 0.2 mm^*^	4.9 ± 0.1 mm^*^	1.2 ± 0.08 mm
Missing teeth (*n*)	8.2 ± 0.5 teeth^*^	9.3 ± 0.8 teeth^*^	2.4 ± 0.4 teeth

* Compared with systemically healthy controls (*P* < 0.05).

### Correlation between hemoglobin A1c and probing depth

**Figure 2:**
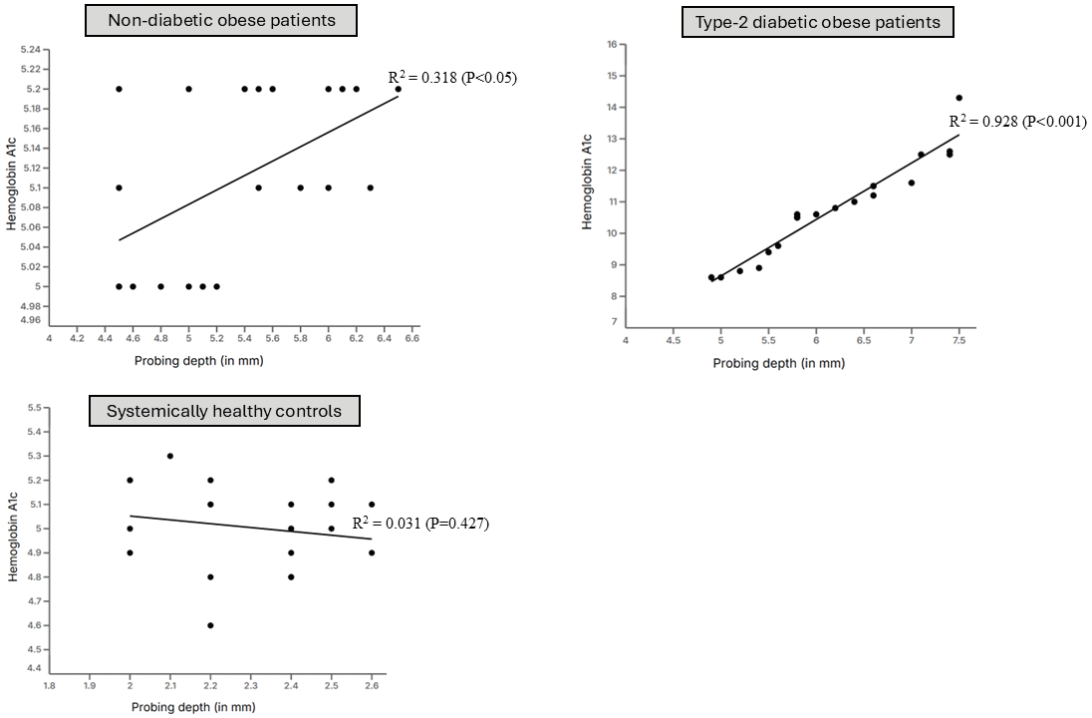
Correlation between hemoglobin A1c and probing depth.

There was a statistically significant correlation between HbA1c and PD among type-2 diabetic (*P* < 0.001) and non-diabetic obese (*P* < 0.05) patients. Among controls, there was no correlation between HbA1c and PD (Figure 2; Table 3). There was no correlation between HbA1c and age, gender, duration of obesity, and type-2 DM, PI, BoP, CAL, and mesial and distal MBL and MT in all groups.

**Table 3:** Correlation between hemoglobin A1c, body mass index and periodontal probing depth.

Groups	Best fit values			95% Confidence intervals			Goodness of fit		*P*-value
	Slope	Y-intercept	X-intercept	Slope	Y-intercept	X-intercept	*R* ^2^	Sy.X	
**Correlation between HbA1c levels and probing depth**									
Non-diabetic obese patients	0.5174 ± 0.03	0.61 ± 0.35	−1.187	0.4484 to 0.58	−0.1384 to 1.36	−3.045 to 0.2363	0.9284	0.318	0.025
Type-2 diabetic obese patients	4.033 ± 1.43	−15.3 ± 7.3	3.78	1.031 to 7.03	−30.58 to 0.09	−0.09080 to 4.34	0.928	0.57	0.0006
Systemically healthy subjects	−0.07812 ± 0.24	2.7 ± 1.24	34.5	−0.6012 to 0.44	0.08360 to 5.31	8.839 to +inf	0.005	0.427	0.427
**Correlation between body mass index and probing depth**									
Non−diabetic obese patients	0.5174 ± 0.03	0.6143 ± 0.35	−1.18	0.4484 to 0.58	−0.1384 to 1.3	−3.045 to 0.23	0.606	0.21	0.0005
Type-2 diabetic obese patients	0.3442 ± 0.15	−5.1 ± 4.9	14.5	0.02 to 0.66	−15.42 to 5.3	−210.9 to 23.2	0.212	0.72	0.01
Systemically healthy subjects	−0.02826 ± 0.04	2.85 ± 0.79	101.01	−0.1127 to 0.05	1.193 to 4.5	40.04 to +inf	0.023	0.2	0.492
**Correlation between body mass index and hemoglobin A1c**									
Non-diabetic obese patients	−0.04 ± 0.02	6.074 ± 0.65	205.4	−0.07150 to 0.01	4.706 to 7.4	104.1 to +inf	0.059	0.08	0.286
Type-2 diabetic obese patients	0.08 ± 0.31	7.98 ± 10.4	−91.95	−0.58 to 0.75	−13.79 to 29.76	−inf to 18.31	0.066	1.52	0.258
Systemically healthy subjects	−0.02 ± 0.03	5.27 ± 0.77	383.9	−0.09 to 0.06	3.63 to 6.9	71.31 to +inf	0.003	0.18	0.931

### Correlation between body mass index and probing depth

**Figure 3:**
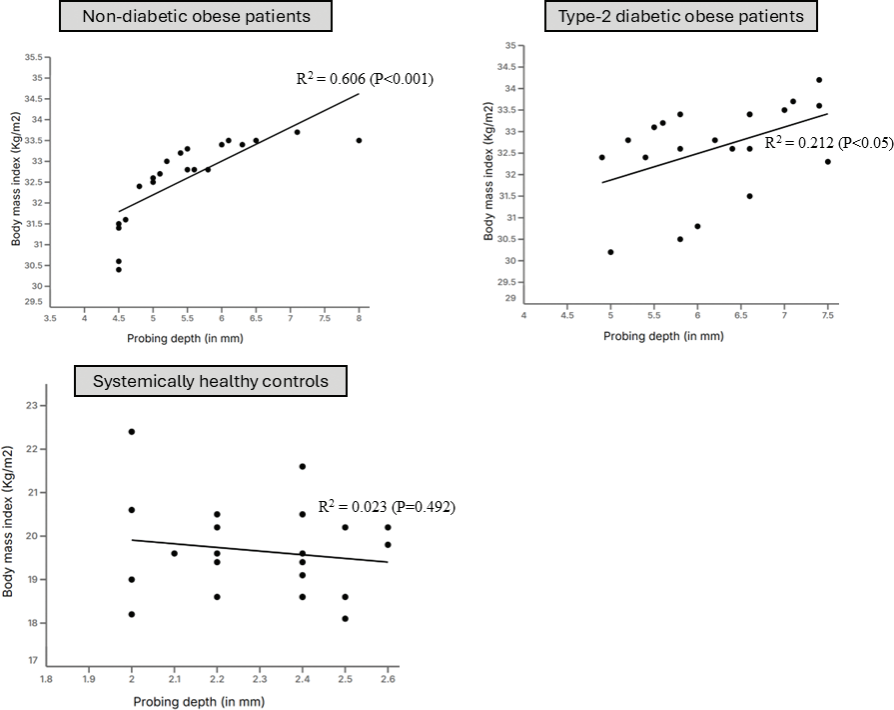
Correlation between body mass index and probing depth.

There was a statistically significant correlation between BMI and PD among type-2 diabetic (*P* < 0.05) and non-diabetic obese (*P* < 0.001) patients. Among controls, there was no correlation between HbA1c and PD (Figure 3; Table 3). There was no correlation between BMI and age, gender, duration of obesity, and type-2 DM, PI, BoP, CAL, and mesial and distal MBL and MT in all groups (Table 4).

**Table 4:** Multiple regression analysis model of factors affecting periodontal health among the study participants.

Predictor variable	Outcome variable	β coefficient	Standard error	95% CI for β	*t*-value	*P*-value
Age	BMI	0.02	0.03	–0.05 to 0.09	0.65	0.52
Gender	BMI	–0.08	0.06	–0.20 to 0.05	–1.28	0.21
Duration of obesity	BMI	0.05	0.04	–0.03 to 0.13	1.15	0.25
Type-2 DM	BMI	0.07	0.05	–0.02 to 0.16	1.44	0.16
PI	BMI	0.03	0.04	–0.05 to 0.11	0.78	0.44
BoP	BMI	0.02	0.03	–0.04 to 0.08	0.67	0.51
CAL	BMI	0.01	0.02	–0.03 to 0.05	0.43	0.67
Mesial MBL	BMI	–0.02	0.03	–0.08 to 0.04	–0.65	0.52
Distal MBL	BMI	–0.01	0.03	–0.07 to 0.05	–0.34	0.74
Missing teeth	BMI	0.04	0.05	–0.06 to 0.14	0.84	0.40
HbA1c	PD	0.21	0.08	0.05 to 0.37	2.63	0.01^*^
BMI	PD	0.18	0.07	0.04 to 0.32	2.47	0.02^*^

BMI: body mass index; CI: confidence interval; DM: diabetes mellitus; MBL: marginal bone loss; BoP: bleeding on probing; PI: plaque index; CAL: clinical attachment loss; HbA1c: hemoglobin A1c.

* Statistical significance.

### Correlation between body mass index and hemoglobin A1c

**Figure 4:**
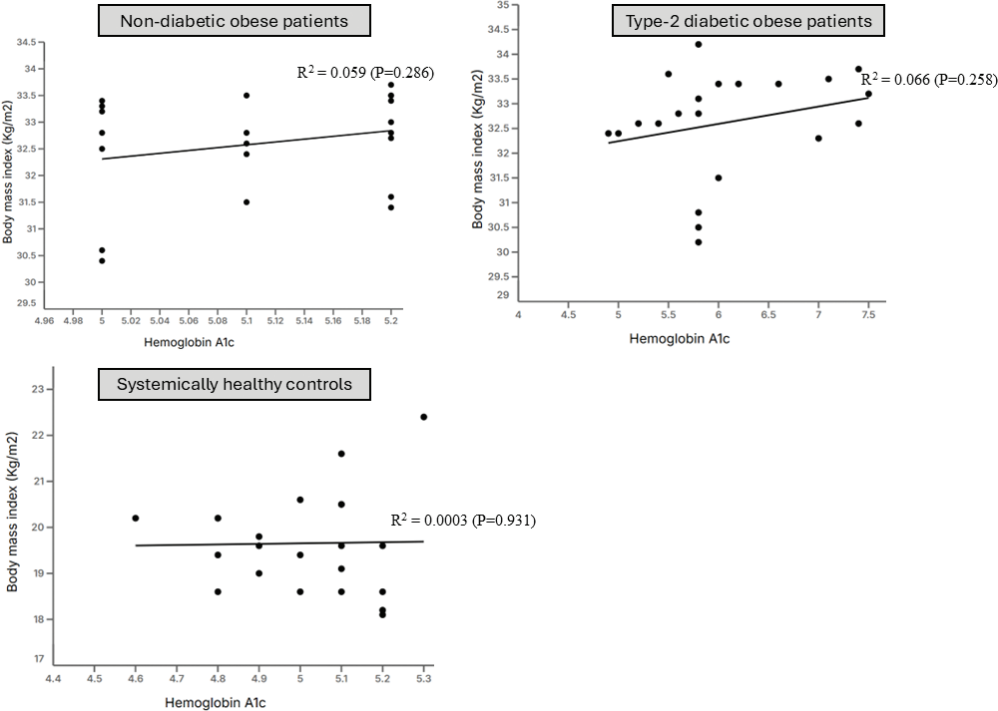
Correlation between body mass index and hemoglobin A1c.

In all groups, there was no association between BMI and HbA1c (Figure 4; Table 3).

## Discussion

The linear regression analysis (LRA) based results showed a statistically-significant correlation between PD and HbA1c and BMI among non-diabetic and type-2 diabetic obese patients. It is worth mentioning that all patients with type-2 DM had poorly-controlled DM with HbA1c levels approximating 11%. Chronic hyperglycemia induces a pro-inflammatory state, increasing cytokines like TNF-α, IL-6, and IL-1β, which enhance periodontal tissue destruction [33, 34]. Moreover, persistent hyperglycemia leads to an increased expression and accumulation of AGEs in periodontal tissues, which impair fibroblast function and collagen synthesis, reducing periodontal tissue repair capacity [35]. On a similar note, a state of obesity by itself is a risk factor of periodontal destruction and it increases the release of adipokines (such as leptin and resistin) and destructive inflammatory cytokines such as IL-6. IL-8, IL-1β and TNF-α [36–38]. It is therefore speculated that with increasing HbA1c and BMI levels, the inflammatory response augments and is clinically reflected as high periodontal probing depths [39]. The radiographic outcomes of the present study demonstrated that both mesial and distal MBL, as well as the number of MT, were significantly greater among obese individuals with and without type-2 diabetes compared with systemically healthy subjects. This finding aligns with earlier studies [6, 38, 40] showing that MBL is more pronounced in patients with obesity and/or diabetes due to the heightened pro-inflammatory milieu and impaired host response associated with these systemic conditions. A recent study [41] evaluated the association between BMI and HbA1c levels among 18–79 years old United States residents. After adjusting the data for confounders, the results showed no statistically significant correlation between BMI and HbA1c levels [41]. However, the present results are in accordance with this study [41] as the LRA found no statistically significant correlation between BMI and HbA1c levels. Notably, all patients in the present study presented with class-I obesity, which may account for the absence of a significant relationship between BMI and HbA1c levels. It is plausible that including patients with class II and class III obesity might reveal a significant correlation between BMI and HbA1c levels. Therefore, further research involving diabetic patients across various obesity classes is warranted to comprehensively assess the relationship between BMI and HbA1c levels.

It is well-known that an underprivileged education status (ES) is a risk factor of poor oral hygiene status and periodontal disease [6, 42]. As shown in Table 1, the ES (university level) of controls was superior compared to type-2 diabetic and non-diabetic patients. In this context, it is also possible that obese patients (type-2 diabetic as well as non-diabetic) demonstrated poor compliance toward their routine OHM. It is notable that none of the non-diabetic and type-2 diabetic patients had ever used dental floss and most of these individuals were brushing teeth once daily. Moreover, these individuals were not routinely visiting oral healthcare providers for dental check-ups and dental prophylaxis. The authors of the present study applaud the study by Newton and Asimakopoulou [1] according to which, poor OHM is a significant risk factor for periodontal disease. It is indeed possible that the inflammatory response induced by hyperglycemia as well as obesity contributed toward augmenting periodontal inflammation in the type-2 diabetic and non-diabetic patients with obesity; however, the contribution of poor routine OHM toward higher scores of PI, BoP, PD, mesial and distal MBL and MT in this context cannot be overlooked. In order to precisely assess the effect of obesity and type-2 DM on periodontal status, additional prospective studies, specifically on patients following stringent oral hygiene practices and routinely visiting oral health care providers for check-ups and routine dental prophylaxis, are needed.

In the present study, all participants had type-1 obesity. The authors applaud the results of a clinical study [40] in which clinical periodontal inflammatory parameters and whole salivary IL-6 and IL-1β were significantly higher among patients with class II and III obesity than patients with class I obesity. Therefore, the severity of periodontal destruction may be higher in patients with class II and III obesity compared with individuals with class 1 obesity. Moreover, the influence of non-surgical periodontal interventions such as SRP has been reported to be effective in reducing merely shallow but not deep periodontal pockets. It is hypothesized that adjunct therapies such as photobiomodulation and/or photodynamic therapy are needed for treating periodontitis in type-2 diabetic and non-diabetic patients with varying classes of obesity. This warrants additional power-adjusted and well-designed clinical trials.

A limitation of the present study is that participants were recruited through CS. In other words, individuals who were readily accessible to the investigators were evaluated. The authors acknowledge that in the present investigation, no systematic probability-based sampling frame was employed, and that selection was not randomized across the underlying target population. While CS can facilitate data collection in a timely and cost-effective manner, it inevitably carries limitations, particularly in terms of external validity and representativeness. Moreover, the waist circumference (WC) and waist-to-height ratios were not measured. Kangas et al. [39] proposed that these parameters are associated with periodontal pocketing in non-diabetic-obese patients. It is perceived that WC and waist-to-height ratio may also be related to the progression of periodontitis in type-2 diabetic patients; however, there are no studies that have tested this hypothesis. Moreover, in the present study, laboratory-based investigations, such as the assessment of hallmark destructive inflammatory cytokines in biological fluids, including whole saliva (WS) and/or gingival crevicular fluid (GCF), have remained uninvestigated. Furthermore, from a microbiological perspective, subgingival plaque samples were not assessed for the presence of periodontopathogenic microbes such as the red complex bacteria. It is hypothesized that the subgingival microbial load of periodontopathogenic bacteria and expression of proinflammatory cytokine in WS and GCF is elevated in type-2 diabetic and non-diabetic obese patients than in systemically healthy controls. It is perceived that the outcomes of periodontal interventions such as SRP are compromised in obese patients with and without type-2 DM than in systemically healthy subjects. Further studies are needed to these hypotheses.

## Conclusion

Periodontal tissue destruction is more pronounced in obese individuals, irrespective of diabetic status. This suggests that excess adiposity plays a central role in periodontal breakdown, regardless of diabetic status.
